# Characterization of Terpene Synthase from Tea Green Leafhopper Being Involved in Formation of Geraniol in Tea (*Camellia sinensis*) Leaves and Potential Effect of Geraniol on Insect-Derived Endobacteria

**DOI:** 10.3390/biom9120808

**Published:** 2019-11-30

**Authors:** Ying Zhou, Xiaoyu Liu, Ziyin Yang

**Affiliations:** 1 Guangdong Provincial Key Laboratory of Applied Botany & Key Laboratory of South China Agricultural Plant Molecular Analysis and Genetic Improvement, South China Botanical Garden, Chinese Academy of Sciences, Xingke Road 723, Tianhe District, Guangzhou 510650, China; yzhou@scbg.ac.cn (Y.Z.); 18423326041@139.com (X.L.); 2 Center of Economic Botany, Core Botanical Gardens, Chinese Academy of Sciences, Xingke Road 723, Tianhe District, Guangzhou 510650, China

**Keywords:** aroma, *Camellia sinensis*, green leafhopper, geraniol, tea, volatile

## Abstract

When insects attack plants, insect-derived elicitors and mechanical damage induce the formation and emission of plant volatiles that have important ecological functions and flavor properties. These events have mainly been studied in model plants, rather than crop plants. Our study showed that tea green leafhopper (*Empoasca* (*Matsumurasca*) *onukii* Matsuda), a major pest infesting tea attack significantly induced the emission of geraniol from tea leaves, but did not affect the crude enzyme activity of geraniol synthase in tea leaves. An enzyme extract of *E. (M.) onukii* specifically produced geraniol from geraniol diphosphate. Furthermore, a terpene synthase (EoTPS) was isolated from *E. (M.) onukii*. This terpene synthase was able to convert geraniol diphosphate to geraniol in vitro. In addition, geraniol had in vitro ability to inhibit the growth of *Acinetobacter johnsonii* that is endobacterial isolated from *E. (M.) onukii*. This information illustrates that elicitors from piercing-sucking insects can induce the formation of volatiles from crop plants and advances our understanding of the roles of plant volatiles in the interaction among crops-insects-microorganisms.

## 1. Introduction

The interaction between insects and plants mediated by plant volatiles has attracted much interest [1,2], and many studies have revealed details of the early and late events related to plant volatile emissions after insect attack [3]. When plants are attacked by insects, mechanical damage is one of the first events. Although simple mechanical damage (single damage, such as one needle sting) may induce a plant response, it may be insufficient to induce the full response to insect attack because insect damage is usually continuous. Therefore, various automatic machines have been designed to simulate continuous wounding by insects [4,5]. Continuous mechanical wounding resembling insect feeding can induce the production of volatiles similar to those induced by insects [4,5]. Another important early event in the interaction between insects and plants is the entry of insect-derived elicitors into plant tissues. The most frequently reported insect-derived elicitors that induce the formation and emission of plant volatiles are fatty acid-amino acid conjugates [6,7]. Other elicitors such as β-glucosidase [8], inceptin [9], caeliferins [6], and an unidentified heat-labile constituent [10] have also been identified and shown to induce the formation and emission of plant volatiles. These elicitors have mainly been identified in chewing insects, and rarely in piercing-sucking insects. This may be because the amount of elicitors produced by small piercing-sucking insects is insufficient for analysis and difficult to obtain [2]. In addition, the first events in the interaction between insects and plants have mainly been studied in model plants, rather than horticultural crop plants.

Tea (*Camellia sinensis*) is an important horticultural crop in almost 30 countries, including China, Japan, India, and Kenya. Abundant volatiles compounds such as fatty acid-derivatives including (*Z*)-3-hexen-1-ol, (*Z*)-3-hexenyl acetate, (*E*)-2-hexenal, and (*E*)-2-hexenoic acid; volatile phenylpropanoids/benzenoids including benzyl nitrile, benzaldehyde, and indole; and volatile terpenes including geraniol, farnesene, ocimenes, linalool, and nerolidol were found in tea [5,11,12,13]. These volatile compounds are important quality components of teas and contribute to tea flavor [14]. Among these volatiles, terpenes are major floral and honey flavor-related volatiles [14]. During tea plant growth, many pest insects such as tea green leafhoppers, tea geometrid, tea aphids, smaller tea tortrix, and Kanzawa spider mites attack tea leaves and affect the yield and quality of the crop [13]. In response to these insect attacks, tea plants emit numerous compounds. Insect-induced volatiles may play important roles in defense [11,12]. On the other hand, insect-induced volatiles may change the flavor the tea and increase the tea quality. As a classical example, a famous oolong tea (oriental beauty), which is manufactured from tea leaves infected with tea green leafhoppers, contains characteristic volatile monoterpenes and has a unique ripe fruit and honey aroma [5,15].

In our previous study, we elucidated the effect of tea green leafhopper (*Empoasca* (*Matsumurasca*) *onukii* Matsuda) attack on the formation and emission of linalool in tea leaves. We found that continuous wounding was a key factor in the formation and emission of linalool from tea leaves exposed to *E. (M.) onukii* attack [5]. In addition, we found that *E. (M.) onukii* attack also significantly induced the emission of geraniol from tea leaves, but did not affect the enzyme activity of geraniol synthase (GES) in tea leaves. However, the reason is unknown. Geraniol is an acyclic monoterpene and contributes to the characteristic floral aroma and flavor in many fruits including *Citrus*, *Vitis vinifera*, and *Litchi chinesis* [16,17,18]. It is also an important floral aroma contributor in tea [14]. To find out whether *E. (M.) onukii* contains GES, *E. (M.) onukii* extracts were analyzed to detect insect-derived elicitors that may be related to the formation of plant volatiles. Moreover, terpene synthase genes from *E. (M.) onukii* were isolated, identified, and functionally characterized. In addition, the effect of geraniol on endobacterial isolated from *E. (M.) onukii* was evaluated. This information will advance our understanding of the roles of plant volatiles in the interaction between horticultural crops-insects-microorganisms, and provide probable evidence that piercing-sucking insect-derived elicitors can induce plant volatiles emission.

## 2. Materials and Methods

### 2.1. Plant Materials and Treatments

Tea (*C. sinensis* cv. Jinxuan) leaves and *E. (M.) onukii* were obtained from the Yingde Tea Experimental Station of the Tea Research Institute, Guangdong Academy of Agricultural Sciences (Yingde City, Guangdong, China).

To exclude effects of other factors from the tea field environment and precisely control the effect of *E. (M.) onukii* attack on tea leaves, we conducted insect treatment experiments in the laboratory. Thirty *E. (M.) onukii* were used to infest 15 shoots of intact tea leaves (one bud and three leaves) for three days. The control was untreated intact tea leaves. The experiments were carried out at 25 °C and 70% relative humidity.

### 2.2. Collection and Analysis of Geraniol

The methods for the collection and analysis of geraniol were described in our published paper [5]. Solid-phase microextraction (SPME, 2 cm—50/30 μm DVB/CarboxenTM/PDMS Stable FlexTM) was used to collect volatiles emitted from tea leaves in a 250 mL glass container at 25 °C for 1 h. Then, the volatiles collected on the SPME were analyzed by gas chromatography-mass spectrometry (GC-MS). The experiments were performed with three replicates.

The GC-MS system comprised a QP2010 SE instrument (Shimadzu Corporation, Kyoto, Japan) equipped with a SUPELCOWAX^TM^ 10 column (30 m × 0.25 mm × 0.25 μm, Supelco Inc., Bellefonte, PA, USA). The GC temperature conditions were as follows: 60 °C for 3 min, increase of 4 °C/min to 150 °C, increase of 30 °C/min to 240 °C, and then hold at 240 °C for 15 min. The splitless mode was used with a splitless time of 1 min, and helium was the carrier gas (flow rate, 1.0 mL/min). The injector temperature was 230 °C. Mass spectrometry was performed in full scan mode (mass range *m*/*z* 40–200). The geraniol authentic standard was purchased from Sigma-Aldrich (Cat. Number 163333, St. Louis, MO, USA). The product was identified by comparing the mass spectra and retention time with standard substance.

### 2.3. Extraction and Assay Activity of Geraniol Synthase from Tea Leaves

The GES extraction and analysis methods were as described elsewhere [19], with a modification. Fresh powdered tea leaf material (1.5 g) was mixed with 5 mL buffer A (pH 7.2, 50 mM HEPES (hydroxyethyl piperazine ethanesulfonic acid) buffer containing 5 mM ascorbic acid, 10 mM MgCl_2_, 5 mM dithiothreitol (DTT), 5 mM sodium bisulfite, 10% glycerol, 0.1% Tween 20, and 5 mM ethylene diamine tetraacetic acid). Then, 0.05 g PVPP (polyvinyl pyrrolidone), and 0.5 mL protease inhibitor solution (0.05 g/mL, Complete^TM^ ULTRA Tablets, Mini, EASYpack Protease Inhibitor Cocktail, Roche, Mannheim, Germany) were added. The mixture was homogenized on ice, completed to 10 mL with buffer A. The homogenate was vortexed for 1 min followed by ultra-sonicated for 10 min at 4 °C. After centrifuge, the resultant supernatant (5 mL) was desalted over a PD-10 column (Sephadex^TM^G-25M, GE Healthcare, Pittsburgh, PA, USA) equilibrated with buffer B (pH 7.2, 25 mM HEPES buffer containing 100 mM KCl and 10% glycerol). The protein was eluted with 5 mL buffer B, and the eluate was collected and used as the crude GES fraction from tea leaves. The experiments were performed in three replicates.

To assay the GES activity, 1 mL crude enzyme solution was mixed with geraniol diphosphate (GPP) (5 μg), and then reacted at 30 °C for 90 min. Then, volatiles were collected on the SPME (50/30 μm, DVB/CAR/PDMS, Stableflex (2 cm), Supelco Inc.) at 42 °C for 30 min. The controls were GPP or enzyme solution alone. The enzyme reaction products were analyzed by GC-MS. Column: SUPELCOWAX^TM^ 10, 30 m × 0.25 mm, film thickness 0.25 μm, Supelco Inc. The carrier gas was helium (flow rate, 1.0 mL/min). The injection port temperature was 230 °C and operated in splitless mode. The splitless time was 1 min. The initial temperature of oven was 60 °C and kept for 3 min, then programmed to 240 °C at 10 °C/min, and held at 240 °C for 10 min. Full scan mode was applied with mass range of *m*/*z* 40–200.

### 2.4. Extraction and Activity Assay of Geraniol Synthase from Empoasca (Matsumurasca) onukii

Twenty *E. (M.) onukii* adults were separated into two parts; head and body. The insect head or body part was crushed to a powder in liquid nitrogen, and then extracted with 250 μL buffer (50 mM Tris-HCl (Ph 7.5), 5 mM DTT, 10 mM MgCl_2_) by ultra-sonication on ice for 10 min. The homogenate was then centrifuged at 12,000 g for 10 min (4 °C). The resultant supernatant (100 μg crude protein) was mixed with GPP (5 μg) in a reaction buffer (50 mM Tris-HCl (pH 7.5), 5 mM DTT, 10 mM MgCl_2_), and then reacted at 30 °C for 30 min for the GES activity assay [20]. After cooling on ice for 30 min, 25 μL of 0.1 mM ethyl n-decanoate (as an internal standard) was added. The reaction products was extracted with 250 μL of hexane:ethyl acetate (1:1, *v*/*v*). The mixture was vortexed for 30 s, followed by centrifuging at 12,000 g for 1 min (4 °C). The extraction step was repeated twice. The combined organic solvent fractions were dried by passage through a glass column packed with anhydrous sodium sulfate. The dried extracts were concentrated to 50 μL under a flow of nitrogen. 1 μL of concentrated extract was subjected to the GC-MS for analysis. Column: SUPELCOWAXTM 10, 30 m × 0.25 mm, film thickness 0.25 μm, Supelco Inc. The carrier gas was helium (flow rate, 1.0 mL/min). The injection port temperature was 230 °C and operated in splitless mode. The splitless time was 1 min. The initial temperature of the oven was 60 °C and kept for 3 min, then programmed to 240 °C at 4 °C/min, and held at 240 °C for 20 min. Full scan mode was applied with mass range of *m*/*z* 40–200. The experiments were performed in three replicates.

### 2.5. Empoasca (Matsumurasca) onukii Terpene Synthase Genes Cloning

Twenty *E. (M.) onukii* was used to isolate total RNA with 1 mL Trizol (Thermo Fishier Scientific, Waltham, MA, USA). One microgram of total RNA was used for first strand reverse transcription with random primers using PrimeScript 1st Strand cDNA Synthesis Kit according to the manufacturer’s protocol (Takara, Dalian, China). A full-length transcriptome of whole *E. (M.) onukii* insect was sequenced by Novogene Company (Tianjin, China; http://www.novogene.com/) with three generation PacBio sequencing technology (unpublished data). The candidate genes were identified in our transcriptome database by blast searches, using the geranylgeranyl diphosphate (GGPP) synthase described in *Nasutitermes takasagoensis* [21] and the terpene synthase described in *Phyllotreta striolata* [22]. The primers were designed with the software Primer premier 5. Nested PCR was applied to amplify the full-length open reading frames (ORFs) with PrimeSTAR Max DNA Polymerase. The primers in Tables S1 and S2 were used for ORFs cloning. The amplified PCR products were cloned into pGEM-T easy vector and sequenced. The protein sequences were aligned, and MEGA software was used to generate neighbor-joining tree [23].

### 2.6. Recombinant Expression of Empoasca (Matsumurasca) onukii Terpene Synthases in Escherichia Coli

The ORFs of target genes were cloned into BamHI/SalI-digested pET-32a vector using an In-fusion HD Cloning Kit (Clontech, Mountain View, CA, USA). The constructed vectors were transformed into *E. coli* BL21 Star (DE3) for protein expression. Cells were induced with 0.1 mM isopropyl-β-D-thiogalactopyranoside at an OD_600_ about 0.6. Additionally, then the cell cultures were incubated at 20 °C for another 16 h. The supernatant that were separated from the cell debris by centrifugation after sonication was incubated with Ni sepharose (GE healthcare, Pittsburgh, PA, USA) for 1 h at 4 °C and then this mixture was loaded to the column for purification according to the manufacturer’s instruction. To remove imidazole, the purified protein was loaded into the PD-10 desalting column equilibrated with 25 mM Tris-HCl for buffer exchange. The protein was eluted with equilibrated buffer and stored at −80 °C until use.

### 2.7. Recombinant Expression of Empoasca (Matsumurasca) onukii Terpene Synthases in Insect Cell Sf9

The putative *E. (M.) onukii* terpene synthases were expressed in Sf9 cell via the Bac to Bac Baculovirus Expression System (Thermo Fishier Scientific). The ORFs of target genes were cloned into BamHI/HindIII-digested pFastBac HTB vector. The constructed vectors or empty vector were transformed into the *E. coli* DH10Bac competent cell (Thermo Fisher Scientific) to generate recombinant bacmid. Sf9 cells was transfected with 1 μg of recombinant bacmid by lipofectimine 2000 (Thermo Fishier Scientific) for 72 h to obtain initial baculoviral stock. After amplifying for three generations, the baculoviral stock was used to infect Sf9 cells for recombinant protein expression. The Sf9 cells were collected and lysed with sonication after infecting for 72 h. The supernatant was used as crud enzyme to assay terpene synthase activity.

### 2.8. Recombinant Empoasca (Matsumurasca) onukii Terpene Synthase Enzyme Assay

The GES activity was evaluated using GPP as a substrate. The reaction volume was 10 mL containing 50 mM Tris HCl (pH 7.5), 10 mM MgCl_2_, 5 mM DTT, 5 μg GPP, 10 μg purified protein (protein expressed by *E.* *coli*), or 100 μg crude protein (protein expressed by Sf9 cells). The reactions were incubated at 30 °C for 90 min [20]. Volatile product was collected by SPME for 30 min at 42 °C. The volatiles absorbed by SPME were analyzed using GC-MS. The geraniol authentic standard was purchased from Sigma-Aldrich (Cat. Number 163333). Column: SUPELCOWAX^TM^ 10 column, 30 m × 0.25 mm, film thickness 0.25 μm, Supelco Inc. The carrier gas was helium (flow rate, 1.0 mL/min). The injection port temperature was 230 °C and operated in splitless mode. The splitless time was 1 min. The initial temperature of oven was 60 °C and kept for 3 min, then programmed to 240 °C at 10 °C/min, and held at 240 °C for 10 min. Full scan mode was applied with a mass range of *m*/*z* 40–200. The experiments were performed in three replicates.

### 2.9. Extraction and Activity Assay of Geraniol Synthase from Other Insects

Two major pests of tea plants and one major pest of sweet potato were used to assay the GES activity of crude enzymes extracted from the insects. These insects were larvae of *Ectropis oblique* and *Euproctis pseudoconspersa*, and adult of *Cylas formicarius*. Briefly, one *E. oblique*/*E. pseudoconspersa* larvae or ten *C. formicarius* adults were homogenized with 250 μL buffer (50 mM Tris HCl, 10 mM MgCl_2_, 5 mM DTT, pH 7.5) on ice. The supernatant was transferred to a new tube and saved as crude enzyme after centrifuge at 12,000 g for 20 min. The reaction volume was 1 mL containing 50 mM Tris HCl (pH 7.5), 10 mM MgCl_2_, 5 mM DTT, 100 μg crude enzyme, and 5 μg GPP. The reaction was incubated at 30 °C for 90 min. The volatile product was collected by SPME for 30 min at 42 °C. The volatiles absorbed by SPME were analyzed using GC-MS. Column: SUPELCOWAX^TM^ 10 column, 30 m × 0.25 mm, film thickness 0.25 μm, Supelco Inc. The carrier gas was helium (flow rate, 1.0 mL/min). The injection port temperature was 230 °C and operated in splitless mode. The splitless time was 1 min. The initial temperature of the oven was 60 °C and kept for 3 min, then programmed to 240 °C at 10 °C/min, and held at 240 °C for 10 min. Full scan mode was applied with a mass range of *m*/*z* 40–200. The experiments were performed in three replicates.

### 2.10. Empoasca (Matsumurasca) onukii Endobateria Isolation, Identification, and Antibacterial Activity Assay of Geraniol

The *E. (M.) onukii* adults were anaesthetized with CO_2_, and then placed on ice. The anaesthetized insects were collected in 1.5 mL eppendorf tube and washed with 70% ethanol for 1 min to kill the external bacterial. Then, the insects were washed with ddH_2_O twice. The washed insects were cut into pieces with dissecting forceps. The dissected insects were placed in a Luria Broth plate and incubated at 30 °C for two days. The bacteria were growing around the insect pieces. The single colonies were further isolated by plate streaking. The genomic DNA was extracted and the bacterial was identified by 16S ribosomal RNA (rRNA) sequencing. DNA was extracted with TIANamp Bacteria DNA kit (Tiangen, Beijing, China) according to the manufacturer’s instruction. The forward primer used to amplify 16S rRNA is 5′-AGTTTGATCMTGGCTCAG-3′. The reverse primer is 5′-GGTTACCTTGTTACGACTT-3′. The length of the sequenced fragment is about 1500 bp. The obtained sequences were blasted in the NCBI database to identify the similarity with other reported species and the 16S rRNA sequences were aligned using ClustalX program with the available sequences. Phylogenetic tree was generated by MEGA with the neighbor-joining method. The statistical robustness of the clusters was evaluated by bootstrap analysis after 1000 replicates.

The isolated endobacteria *Acinetobacter johnsonii* was used for antibacterial activity assay of geraniol. The geraniol was dissolved in Tween 20. The final concentration of geraniol in LB medium was 0, 100, 250, and 500 μg/mL, respectively. The final concentration of Tween 20 in the LB medium was 1%. One hundred microliters of the exponentially growing bacterial cells (OD_600_ = 0.3) was inoculated into 10 mL LB medium containing geraniol with different concentration. Then, the cultures were incubated at 30 °C for another 6 h. Then, OD_600_ was analyzed to evaluate the antibacterial activity.

### 2.11. Statistical Analyses

The statistical difference between the two groups was determined with *t*-test (Paired and independent samples). The *p*-value of less than 0.05 was considered significant. Statistical analyses were performed using SPSS Version 18.0 software (SPSS Inc. Chicago, IL, USA).

## 3. Results

### 3.1. Empoasca (Matsumurasca) onukii Attack Significantly Increased Geraniol Emission from Tea Leaves

**Figure 1:**
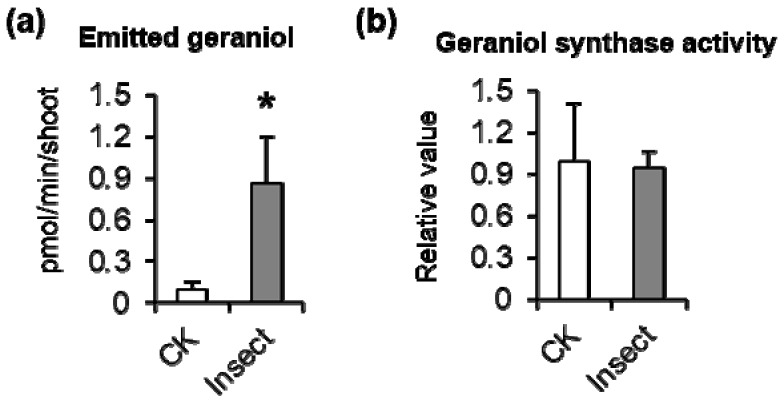
Effect of *Empoasca* (*Matsumurasca*) *(E. (M.)) onukii* attack on emission of geraniol and geraniol synthase activity in tea leaves. (**a**) Effect of *E. (M.) onukii* attack on emission of geraniol in tea leaves in lab control experiments. (**b**) Effect of *E. (M.) onukii* attack on geraniol synthase activity. The control (CK) was intact tea leaves without treatment. The levels of enzyme activity in the control were set as 1. Student *t* test was used to determine the differences between control and treatment. Data are expressed as mean ± S.D. (n = 3) *, *p* ≤ 0.05.

*Empoasca (Matsumurasca) onukii* is the corrected scientific name of tea green leafhopper in China, one of the main pests in tea production regions [24]. In tea fields, *E. (M.) onukii*-infested tea leaves show changes in appearance. Here, we investigated the effect of *E. (M.) onukii* infestation on the formation and emission of geraniol, a volatile monoterpene derived from GPP. We conducted these experiments in the laboratory to exclude the effects of other factors (e.g., other insects/climatic conditions). Compared with control tea leaves, those infested by *E. (M.) onukii* showed a significant increase in geraniol emission (Figure 1a). However, infestation by *E. (M.) onukii* did not affect the activity of GES in tea leaves (Figure 1b).

### 3.2. Crude Enzyme Extract of Empoasca (Matsumurasca) onukii Produced Geraniol from Greanyl Diphosphate

**Figure 2:**
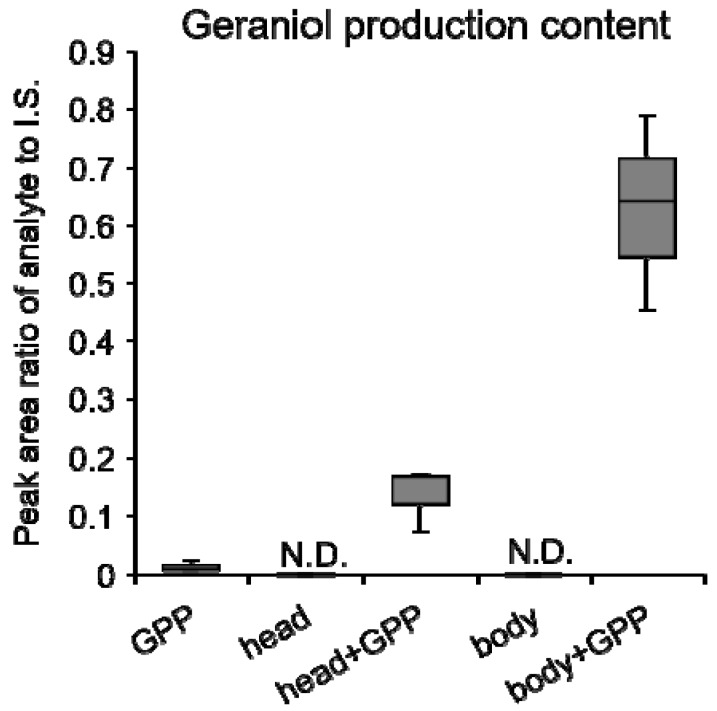
Evaluation of enzyme activity of extract of *E. (M.) onukii* against GPP. Twenty *E. (M.) onukii* adults were separated into two parts; head and body. The head or body part extract reacted with GPP. The controls were GPP or crude extract only. The geraniol production amount was represented by the GC-MS peak area ratio of *m*/*z* 69 (geraniol characteristic ion) to *m*/*z* 88 (I.S. (internal standard, ethyl *n*-decanoate) characteristic ion). N.D., lower than detection limit. Data are expressed as mean ± S.D. (n = 3).

Since the activity of tea leaves GES was not increased after *E. (M.) onukii* attack, it was presumed that the geraniol accumulation may induced by GES which was from *E. (M.) onukii*. To investigate whether elicitors such as enzymes involved in geraniol formation were present in *E. (M.) onukii*, we extracted *E. (M.) onukii* using buffer, and added GPP as the substrate. In contrast to controls (GPP or crude extract only), the reaction solution containing GPP and the insect extract specifically produced geraniol, but not other volatile monoterpenes such as linalool. The GES activity was higher in the body part than in the head part of *E. (M.) onukii* (Figure 2, Table S3). These results confirmed that *E. (M.) onukii* contains GES, which generates geraniol from GPP.

### 3.3. Terpene synthase from Empoasca (Matsumurasca) onukii Was Able to Convert Greanyl Diphosphate to Geraniol

**Figure 3:**
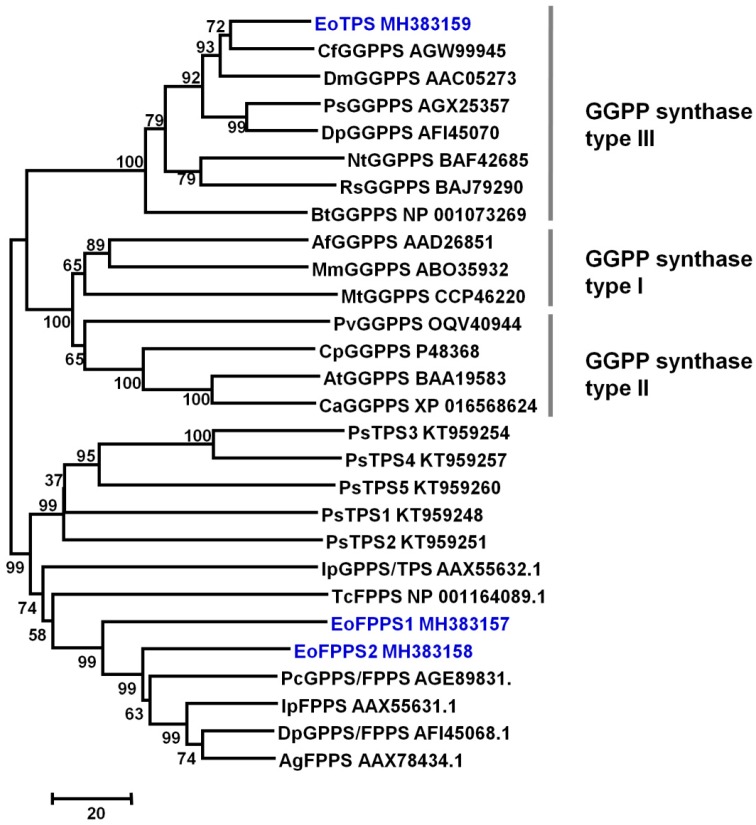
Phylogenetic analysis of terpene synthases in insects. Cf, Choristoneura fumiferana; Dm, Drosophila melanogaster; Ps, Pissodes strobe; Dp, Dendroctonus ponderosae; Nt, Nasutitermes takasagoensis; Rs, Reticulitermes speratus; Bt, Bos Taurus; Af, Archaeoglobus fulgidus; Mm, Methanococcus maripaludis; Mt, Mycobacterium tuberculosis; Pv, Pantoea vagans; Cp, Cyanophora paradoxa; At, Arabidopsis thaliana; Ca, Capsicum annuum; Ps, Phyllotreta striolata; Ip, Ips pini; Tc, Tribolium castaneum; Pc, Phaedon cochleariae; Dp, Dendroctonus ponderosae; Ag, Anthonomus grandis. Bootstrap values from 1000 replicates were used to assess the robustness of the trees.

**Figure 4:**
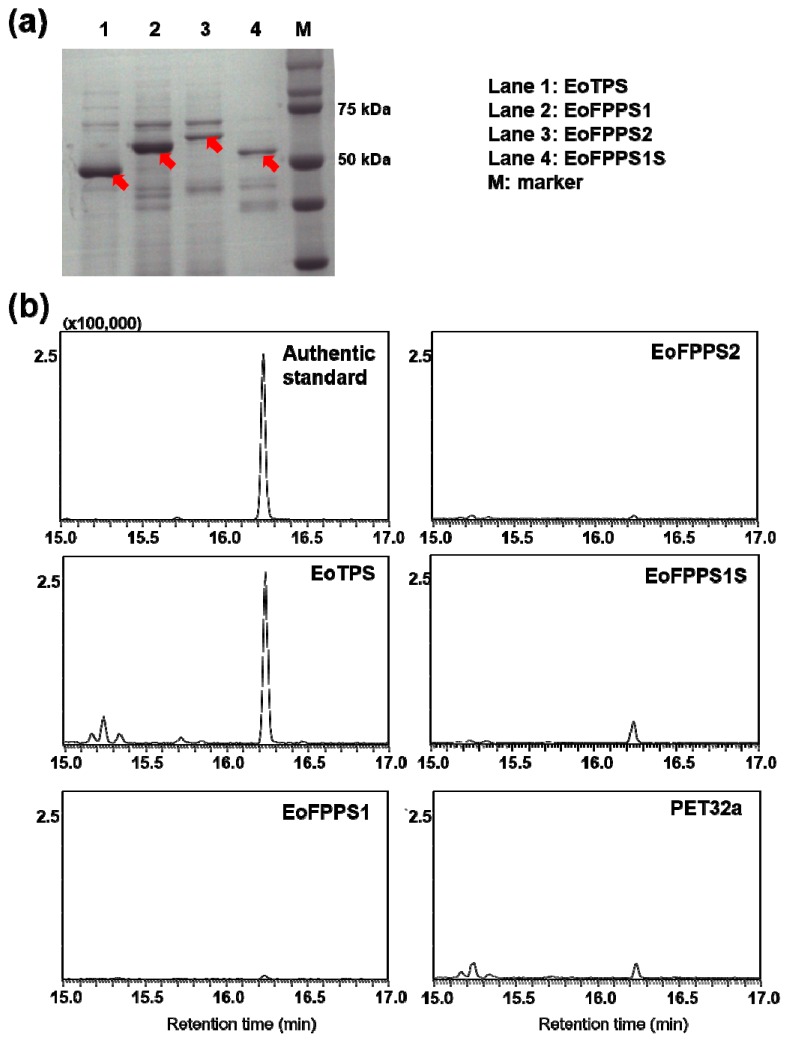
Sodium dodecyl sulfate-polyacrylamide gel electrophoresis (SDS-PAGE) analysis and functional characterization of terpene synthases in *E. (M.) onukii* expressed in *E. coli.* (**a**) SDS-PAGE of purified recombinant putative terpene synthases. EoFPPS1S was the protein lacking putative mitochondrial targeting peptide of EoFPPS1. (**b**) Gas chromatography-mass spectrometry (GC-MS) identification of formation of geraniol from GPP under the actions of recombinant terpene synthases. The experiments were performed in three replicates with similar results. Authentic standard: Geraniol authentic standard.

**Figure 5:**
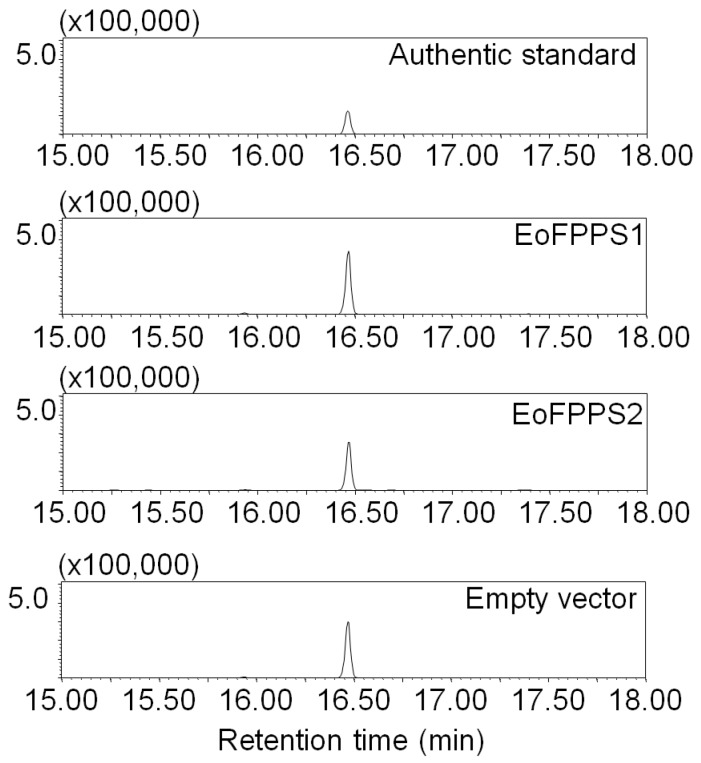
Functional characterization of *E. (M.) onukii* terpene synthases expressed in insect cell Sf9. The formation of geraniol was identified by GC-MS. The formation of geraniol is not different between EoFPPS1 and empty vector or EoFPPS2 and empty vector. The experiments were performed in three replicates with similar results. Authentic standard: Geraniol authentic standard.

Although typical terpene synthase was not found in insect genomic and transcriptomic sequences [22], the isoprenyl diphosphate synthases (IDSs) such as terpene synthases have been reported in *Phyllotreta striolata* and *Ips pini* [22,25]. A full-length transcriptome of whole *E. (M.) onukii* insect was sequenced with three generation PacBio sequencing technology (unpublished data). Three isoprenyl diphosphate synthases including two farnesyl diphosphate (FPPS, *EoFPPS1* and *EoFPPS2*) homologue genes and one geranylgeranyl diphosphate synthase (GGPPS, *EoTPS*) homologue genes were found in this full-length transcriptome. These three genes were cloned from *E. (M.) onukii* successfully. The full length of EoFPPS1 (GenBank Accession Number MH383157) was 1179 bps, encoding 392 amino acids. The full length of EoFPPS2 (GenBank Accession Number MH383158) was 1287 bps, encoding 428 amino acids. The full length of EoTPS (GenBank Accession Number MH383159) was 912 bps, encoding 303 amino acids. The calculated molecular masses of EoFPPS1, EoFPPS2, and EoTPS were 45, 49, and 35 kDa, respectively. Neighbor-joining tree was generated with other isoprenyl diphosphate synthases in insect. The two farnesyl diphosphate synthase, EoFPPS1 and EoFPPS2, belong to the FPP synthase family. They clustered together with the other characterized FPPS from beetles in phylogenetic trees and were closest to the *Phaedon cochleariae* FPPS (Figure 3). The geranylgeranyl diphosphate synthase, EoTPS, belongs to the GGPP synthase type III family (Figure 3) and it was closest with the *Choristoneura fumiferana* GGPPS in the phylogenetic analysis. These genes were heterologously expressed in *Escherichia coli* (Figure 4a). Among these three genes, EoFPPS1 was predicted to be mitochondrion localized. To better determine the enzymatic activity of EoFPPS1, the ORF lacking pupative mitochondrial targeting peptide (the first 25 amino acids, EoFPPS1S) was also expressed in *E. coli* (Figure 4a). The activity assay results showed that only EoTPS converted GPP to geraniol (Figure 4b, Figure S1). It was presumed that prokaryotic expression systems may not be suitable for functional protein expression. Therefore, EoFPPS1 and EoFPPS2 were also expressed in insect Sf9 cells. The results showed that there is no difference of geraniol formation between EoFPPS1 and empty vector, or between EoFPPS2 and empty vector. Therefore, the enzyme activity assay with insect Sf9 cell expression system also proved that EoFPPS1 and EoFPPS2 could not convert GPP to geraniol (Figure 5).

### 3.4. Geraniol Synthase Also Existed in Other Insects

**Figure 6:**
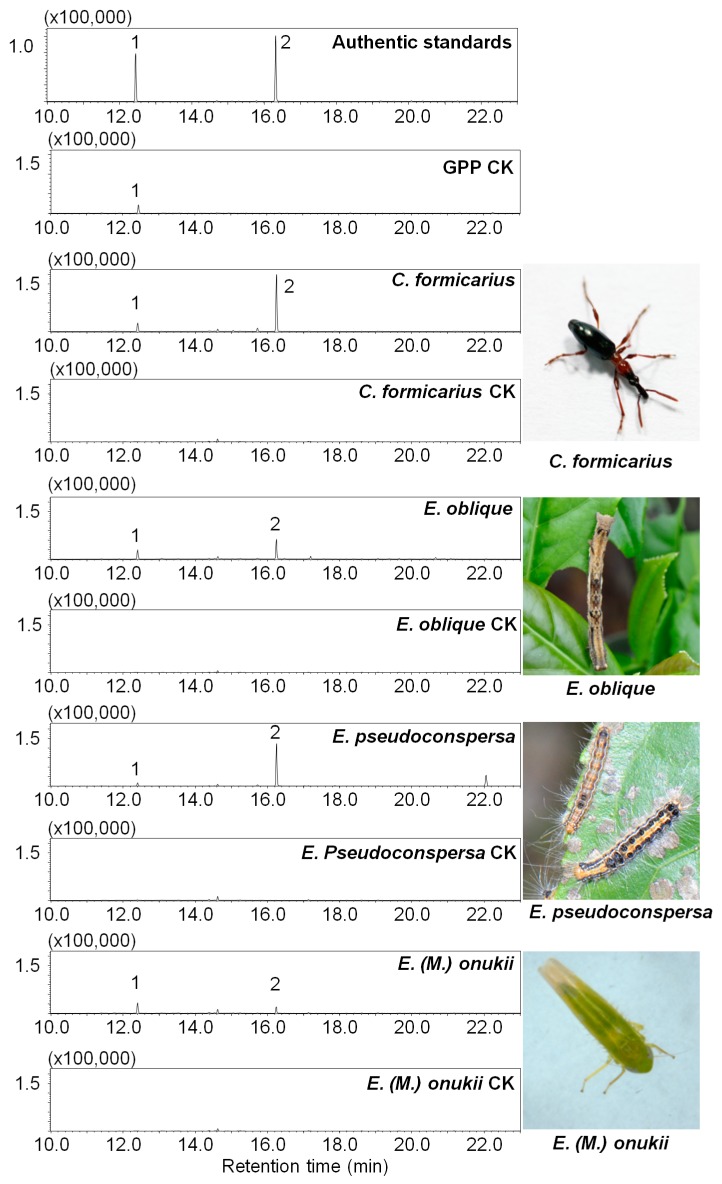
Evaluation of terpene synthases in other insects. GPP reacted with crude enzymes extracted from different insects. *Ectropis oblique*, larvae; *Euproctis pseudoconspersa*, larvae; *Cylas formicarius*, adult; *E. (M.) onukii*, adult. The controls were GPP or crude enzyme only. The formation of linalool in each enzyme activity came from the GPP self-degradation. 1. Linalool; 2. geraniol. GPP CK: GPP alone in the reaction buffer; each insect CK: Crude enzyme alone in the reaction buffer. The experiments were performed in three replicates with similar results.

To identify whether GES was conserved in insects, the GES activity was also determined in other insects including larvae of *Ectropis oblique* and *Euproctis pseudoconspersa*, and adult of *Cylas formicarius*. The activity was measured with crude enzyme extracted from different insects. The monoterpene synthase assay results showed that only geraniol was produced using GPP as a substrate (Figure 6). No significant difference was observed in linalool formation between enzyme reaction and GPP self-degradation control (Figure 6). These results suggested that GES was the monoterpene synthase that existed in these insects.

### 3.5. Geraniol Had in Vitro Ability to Inhibit the Growth of Endobacterial Isolated from Empoasca (Matsumurasca) onukii

**Figure 7:**
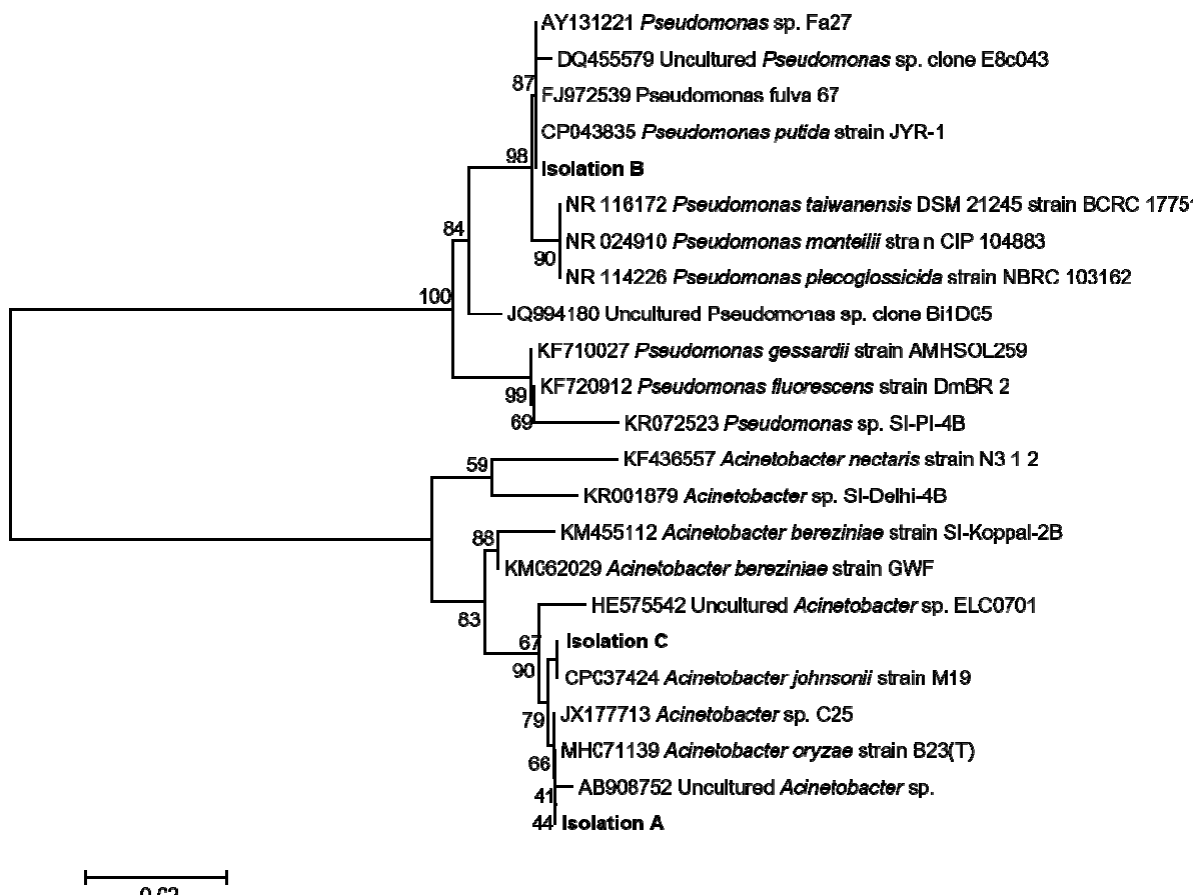
Phylogenetic analysis of the isolated endobacteria. The 16S ribosomal RNA (rRNA) sequences were aligned with ClustalX and neighbor-joining tree were created with MEGA software. Bootstrap values were calculated for 1000 replicates.

To isolate the endobacteria in *E. (M.) onukii*, the dissected insect pieces were incubated in LB plate until the bacteria colonies appeared. Then, the grown bacterial was further purified to obtain single colony. The bacteria DNA was extracted and the 16S rRNA gene was amplified and sequenced. For preliminary identification, the 16S rRNA sequences were analyzed with BLASTn in GenBank database. Isolation A showed similarity of 99.86% with uncultured *Acinetobacter* sp. (AB908752). Isolation B showed similarity of 99.79% with *Pseucomonas fulva* (FJ972539). Isolation C showed similarity of 99.79% with *Acinetobacter johnsonii* (CP037424). In the next steps, these three 16S rRNA sequences were aligned with other sequences of different species in GenBank database. The neighbor-joining phylogenetic tree was constructed. The similar results were found in phylogenetic analysis (Figure 7). These endobacteria were also found in other insects [26,27,28,29]. *Acinetobacter* group was reported to be the major endobacteria in other insects [26]. *Acinetobacter johnsonii* is a common endosymbiont in insect [27,28]. Therefore, in our present study, isolation C *Acinetobacter johnsonii* was chosen to initially analyze the potential antibacterial activity of geraniol. The results showed that geraniol could inhibit the isolation C growth in vitro. The geraniol concentration of 500 μg/mL inhibited the isolation C growth in LB medium effectively according to the OD_600_ analysis (Table 1).

The isolated C colony *Acinetobacter johnsonii* was used for antibacterial activity assay. The experiments were performed in three replicates.

## 4. Discussion

To be widespread volatile, the function of geraniol in plants has been well studied. It has been shown to induce apoptosis-like cell death as a defense reaction against bacterial infection [30,31,32] and to play roles in attracting beneficial insects such as sarcophagid flies and braconid wasps [33]. In tea plants, geraniol is one of the herbivore-induced volatiles in aphid-damaged tea plants [11]. Our study confirmed that the emitted geraniol would be induced by *E. (M.) onukii* attack. Geraniol was reported to be the symbolic aroma which could be used to differentiate different black tea [34]. To be the important flavor-related volatile, the induced geraniol by insect may affect the tea flavor. The pathway of geraniol biosynthesis has been elucidated; it is an acyclic monoterpene alcohol that is synthesized from GPP, the universal precursor of all monoterpenes. The GES from sweet basil was the first GES to be isolated, purified, and functionally characterized. It was shown to generate geraniol from GPP, and to be localized exclusively or almost exclusively to glands [35]. Since then, GESs have been identified and characterized from the evergreen camphor tree *Cinnamomum tenuipilum* [36], perilla [37,38], and Madagascar periwinkle [39]. However, GES had not been isolated from tea leaves.

Terpenes are structurally diverse natural products that have diverse functions in the ecological interactions of many living organisms. Terpenes are biosynthesized by terpene synthases, which have been widely studied in plants and eukaryotic fungi. Recently, terpene synthases in insects have attracted the interest of researchers. The terpene synthase in insect was found to be an evolutionarily novel family, and the sesquiterpene aggregation pheromone synthase was identified in *Phyllotreta striolata* [22]. Most reported terpene synthases in insects are sesquiterpene synthases, while monoterpene synthases are rare. As one of monoterpene, geraniol has been found texist in many insects [40,41,42,43,44]. In the larvae of chrysomelid beetles, geraniol was presumed to be synthesized in fat body [43]. It could serve as a precursor of defense compound called iridoids [41,42]. Moreover, geraniol has been proven to be the active component in Nassanoff glands to attract each other in honey bees [40]. It was also reported as caste determining compound from labial glands in *Melipona* bees [44]. Although these important roles of geraniol had been reported, GES was not identified in the insect. In the present study, GES, a monoterpene synthase specifically producing geraniol, was extracted from *E. (M.) onukii* (Figure 2). Moreover, GES was found to exist in many insects in the present study (Figure 6). These observations indicate that not only sesquiterpene synthases but also monoterpene synthases occur more widely among eukaryotes than previously thought. The higher crude enzyme activity of GES was found in the body part (Figure 2), which indicated that the GES protein may be mainly located in fat body as previously reported [43] or digested system. Keeling [45] reported that the mevalonate pathway genes in midgut were upregulated after phloem feeding, which may be responsible for monoterpenoid pheromone component ipsdienol de novo synthesis. Our study further identified a TPS gene *EoTPS* in *E. (M.) onukii*, which could produce geraniol with GPP as substrate in vitro. Different from those reported terpene synthases in insect, which were all FPPS like enzymes [22,25], our study proved that the GGPPS homologue in *E. (M.) onukii* could function as monoterpene synthase and produce geraniol from GPP. This is the first GGPPS like terpene synthase reported in insects.

Wounding or elicitors from insects induce many events in plants. Insect elicitors such as fatty acid-amino acid conjugates [7,46], inceptin [9], caeliferins [6], and an unidentified heat-labile constituent [10] do not directly participate in the biosynthesis of plant defensive metabolites, but simulate the upstream signals of biosynthesis of plant defensive metabolites. For example, insect elicitors may resemble phytohormones that can activate the genes involved in the formation of plant volatiles, thus leading to the increased emission of plant volatiles [3]. Only β-glucosidase [8] was reported to directly react with glycosidically bound volatiles in plants to release free volatiles. The insect-derived GES detected in this study may directly contact volatile precursors such as GPP to produce free volatiles. Since the activity of GES in tea leaves was not significantly affected by the *E. (M.) onukii* attack, the *E. (M.) onukii*-derived GES may be the main contributor to geraniol emission during insect attack. The further experiments, such as RNAi of EoTPS, would be performed to confirm it. Why did *E. (M.) onukii* stimulate the geraniol emission itself? Additionally, is there any benefit for *E. (M.) onukii* to induce geraniol emission? It has been reported that the microbiome plays important roles in the interaction of plants and herbivores [47,48]. To discover the ecological function of EoTPS-induced geraniol, the endobacteria of *E. (M.) onukii* was isolated and identified by 16S rRNA sequencing (Figure 7). The antibacterial assay with endobacteria of isolation C *Acinetobacter johnsonii* showed that geraniol could inhibit the growth of this bacteria (Table 1), which suggested that the EoTPS-induced geraniol might affect the *E. (M.) onukii* by regulating its endobacteria. Geraniol is a wide-spread volatile in tea (*C. sinensis*). In addition to its great contribution to tea flavor, geraniol is also reported to be a defensive secondary metabolite in plant [49,50,51]. It has been demonstrated to be effective in repelling insects and apparently possessed insecticide properties [52,53]. Geraniol also showed well antipathogenic activity to both plant and insect pathogens [50,51]. Insect endosymbionts play an important role in insect environmental adaption. These microbiotas help insect absorb nutrition [54], metabolize the plant defensive metabolites [55], and mediate the insect reproduction [56]. Although the function of endobacteria *Acinetobacter johnsonii* in *E. (M.) onukii* is still unknown. Considering that some *Acinetobacter* could contribute to insecticide resistance of pest [57], it is hypothesized that geraniol might affect the *E. (M.) onukii* physiological activity by mediating the growth of its endobacteria. Further in situ studies need be performed to prove this hypothesis.

## 5. Conclusions

The most frequently reported insect-derived elicitors that induce the formation and emission of plant volatiles are identified in chewing insects, and rarely in piercing-sucking insects. In the present study, crude enzyme of *E. (M.) onukii* showed specific geraniol synthase activity. A *E. (M.) onukii* geraniol synthase EoTPS was then identified and it could convert GPP into geraniol in vitro. It was presumed that EoTPS might be delivered into tea leaves and induce the geraniol emission during insect attack. How the geraniol affects *E. (M.) onukii* endobacteria and the ecological function of induced geraniol for *E. (M.) onukii* need further study.

## Figures and Tables

**Table 1:** Antibacterial activity assay of geraniol.

Geraniol Concentration (μg/mL)	OD_600_
0	>3
100	>3
250	11.980 ± 0.232
500	0.018 ± 0.006

## Supplementary Materials

The following are available online at https://www.mdpi.com/2218-273X/9/12/808/s1. Table S1: Primers used for gene cloning in first-round PCR. Table S2: Primers used for gene cloning in second-round PCR. Table S3: Detailed data points of Figure 2. Figure S1: Functional characterization of terpene synthases in *E. (M.) onukii* expressed in *E. coli* with three replicates.
