# Supplementary material for: Characterization of Terpene Synthase from Tea Green Leafhopper Being Involved in Formation of Geraniol in Tea (Camellia sinensis) Leaves and Potential Effect of Geraniol on Insect-Derived Endobacteria
Source: Biomolecules. 2019 Nov 30;9(12):808. doi: 10.3390/biom9120808 (PMC6995508; doi:10.3390/biom9120808)
Supplement: Supplementary file 1 [file biomolecules-09-00808-s001.pdf]

Supplementary Materials

Characterization of Terpene Synthase from Tea Green Leafhopper Being Involved in Formation of Geraniol in Tea (*Camellia sinensis*) Leaves and Potential Effect of Geraniol on Insect-Derived Endobacteria

Ying Zhou [1,†], Xiaoyu Liu [1,†] and Ziyin Yang [1,2,*]

[1] Guangdong Provincial Key Laboratory of Applied Botany & Key Laboratory of South China Agricultural Plant Molecular Analysis and Genetic Improvement, South China Botanical Garden, Chinese Academy of Sciences, Xingke Road 723, Tianhe District, Guangzhou 510650, China; yzhou@scbg.ac.cn (Y.Z.); 18423326041@139.com (X.L.)

[2] Center of Economic Botany, Core Botanical Gardens, Chinese Academy of Sciences, Xingke Road 723, Tianhe District, Guangzhou 510650, China

* Correspondence: zyyang@scbg.ac.cn; Tel.: +86-20-38072989

† These authors have contributed equally to this work.

Table S1. Primers used for gene cloning in first-round PCR.

Gene	Accession number	Forward primer 5′-3′	Reverse primer 5′-3′
EoFPPS1	MH383157	AAAGTGCGGACTGGGAGG	AAAGTTATGGGAAGTTGCTG
EoFPPS2	MH383158	ATTGTGGTGAAAACCCTT	CTGTCGTTACATTCGTTAGTT
EoTPS	MH383159	TTGTAAGTTATTAAATCCAT CGCTAC	TTTCCATCCTTTGGTTCTGC

Table S2. Primers used for gene cloning in second-round PCR.

Gene	Accession number	Forward primer 5′-3′	Reverse primer 5′-3′
EoFPPS1	MH383157	TATCGGATCCGAATTCATGA GGGCTAACATGTGGAGG	CCGCAAGCTTGTCGACTTAG CGACCAGTCATGGTCTTGA
EoFPPS2	MH383158	TATCGGATCCGAATTCATGT TCAGCTCTTTAAAACTCAGT C	CCGCAAGCTTGTCGACTTAC GATTCTCGTTTGTAAATCGTG
EoTPS	MH383159	TATCGGATCCGAATTCATGG AGGGGCTCGTCAATAACTC	CCGCAAGCTTGTCGACTTAC TTGGTACGGTCCCAGTTG
